# Hydrophobicity identifies false positives and false negatives in peptide-MHC binding

**DOI:** 10.3389/fonc.2022.1034810

**Published:** 2022-11-07

**Authors:** Arnav Solanki, Marc Riedel, James Cornette, Julia Udell, George Vasmatzis

**Affiliations:** ^1^ Department of Electrical and Computer Engineering, University of Minnesota, Minneapolis, MN, United States; ^2^ Department of Mathematics, Iowa State University, Ames, IA, United States; ^3^ Biomarker Discovery Group, Mayo Clinic, Center for Individualized Medicine, Rochester, MN, United States

**Keywords:** MHC class I, peptide, hydrophobicity, machine learning, neural networks

## Abstract

Major Histocompability Complex (MHC) Class I molecules allow cells to present foreign and endogenous peptides to T-Cells so that cells infected by pathogens can be identified and killed. Neural networks tools such as NetMHC-4.0 and NetMHCpan-4.1 are used to predict whether peptides will bind to variants of MHC molecules. These tools are trained on data gathered from binding affinity and eluted ligand experiments. However, these tools do not track hydrophobicity, a significant biochemical factor relevant to peptide binding, in their predictions. A previous study had concluded that the peptides predicted to bind to HLA-A*0201 by NetMHC-4.0 were much more hydrophobic than expected. This paper expands that study by also focusing on HLA-B*2705 and HLA-B*0801, which prefer binding hydrophilic and balanced peptides respectively. The correlation of hydrophobicity of 9-mer peptides with their predicted binding strengths to these various HLAs was investigated. Two studies were performed, one using the data that the two neural networks were trained on, and the other using a sample of the human proteome. NetMHC-4.0 was found to have a statistically significant bias towards predicting highly hydrophobic peptides as strong binders to HLA-A*0201 and HLA-B*2705 in both studies. Machine Learning metrics were used to identify the causes for this bias: hydrophobic false positives and hydrophilic false negatives. These results suggest that the retraining the neural networks with biochemical attributes such as hydrophobicity and better training data could increase the accuracy of their predictions. This would increase their impact in applications such as vaccine design and neoantigen identification.

## 1 Introduction

The Human Leukocyte Antigen (HLA) gene system encodes cell-surface proteins that play a key role in the immune system. HLA proteins of Major Histocompatibility Complex (MHC) Class I allow nucleated cells to present peptides from within the cell (1). In these cells, endogenous proteins are eventually broken down into small peptides, 8-15 amino acids long, by the proteasome. These antigens are then trafficked to and loaded onto MHC Class I molecules. If sufficient binding affinity is achieved then a stable peptide-MHC (pMHC) complex is formed and transported to the cell surface. Self-peptides, antigens encoded in the human proteome, and foreign peptides, derived from pathogenic proteins, can thus be presented. By surveilling these extracellular pMHCs, CD8^+^ T-cells can distinguish normal cells from pathogen-infected cells, and kill the latter.

The mechanics of peptide binding are specific to a given MHC variant. The HLA genes are among the most diverse in the human population  (2). Thus the set of all antigens presented by a person’s MHCs, labelled as their *immunopeptidome*, is unique and determines the capacity of their immune system. Since the immune response of a person to a viral infection like COVID-19, for instance, is dependent on whether the foreign antigens presented by their MHCs are distinguishable from self-peptides, understanding and predicting pMHC binding is an important topic. In this paper, we have focused on NetMHC-4.0  (3) and NetMHCpan-4.1 (4), two state-of-the-art neural network based methods that predict pMHC binding. Both software tools have been applied in predicting cancer immune escape mechanisms (5), checkpoint blockade immunotherapy for tumors (6), and identifying COVID-19 T-cell response targets (7).

While these tools provide valuable pMHC predictions, they do not model pMHC binding at the molecular level or capture the entire antigen presentation pathway’s effects. Hydrophobicity is a measure of how repulsive a molecule is to water, often a consequence of nonpolarity. It plays a vital role in protein binding – for example, the MHC molecule HLA-A*0201 (A2) contains hydrophobic binding pockets that bind to correspondingly hydrophobic amino acids (8). In contrast, the MHC HLA-B*2705 (B27) prefers to bind peptides with a hydrophilic amino acid in one of its pockets (9). Historically, immunopeptidomes have been predicted by modelling the interaction of the MHC binding pocket and peptide, particularly focusing on biochemical attributes such as sidechain conformations, solvation energies, electrostatic interactions, and hydrophobicity (10, 11). However with improved computing power, larger datasets, and the need for interpolation due to the high polymorphism in MHC Class I alleles (12), artificial intelligence based methods have become popular over such mechanistic means of prediction. As NetMHC-4.0 and NetMHCpan-4.1 are trained with sequence data and binding scores only, they lack the means of modelling these biochemical attributes. Other software tools such as ANN-Hydro  (13) have utilized hydrophobicity in their immunogenic predictions, but do not predict binding affinity and are outperformed by NetMHCpan (14). In our use of NetMHC-4.0 we had observed a prevalence of highly hydrophobic peptides in the predicted A2 immunopeptidome. We had found this contrary to our expectations, since peptides in which all amino acids are hydrophobes would not dissolve in the aqueous cytosol within the cell and would thus likely not be available for binding with the MHC. We had therefore sought to investigate the possibility that these tools were over-estimating binding scores for such hydrophobic peptides. In a previous study (15), we had tested these tools’ predictions on A2 and observed hydrophobic biases that suggested a false positive problem in NetMHC-4.0. Here, we expanded that study to look at multiple HLAs with different binding preferences in more detail. Once again, we conducted two analyses on both NetMHC-4.0 and NetMHCpan-4.1, one using training data and the other using a sample of the human proteome, to investigate the correlation of predicted strong binders and hydrophobicity. We present our results and highlight the unintended bias within NetMHC-4.0 for predicting hydrophobic peptides as strong binders, and for predicting hydrophilic peptides as non-binders.

## 2 Methods

NetMHC-4.0 and NetMHCpan-4.1 allow users to input a list of peptides or whole proteins, and test the binding of all peptides with a chosen MHC molecule. Both tools return an adjusted score between 0 (for non-binders) and 1 (for strong binders) for all peptides. A notable distinction between the two is that NetMHC is limited to predicting binding for MHC variants it is trained on, i.e. curated MHCs. In contrast, NetMHCpan is capable of interpolating predictions for uncurated MHCs if users provide the MHC amino acid sequence. This is achieved through the integration of MHC sequence as a data feature in training, and by a larger training dataset generated using a sophisticated machine learning method called NNAlign_MA (16). NetMHCpan-4.1 consists of an ensemble of 50 neural networks, each with hidden layers containing 55 and 66 neurons, that were trained using 5-fold cross validation. NetMHC-4.0 consists of 20 neural networks, each with a single hidden layer of 5 neurons, that were trained using a nested 5-fold cross validation approach (3).

### 2.1 Data mining

NetMHC-4.0 was trained on CD8^+^ epitope binding affinity (BA) data from the Immune Epitope Database. This data provides binding scores for peptides to single allele MHCs, with a score that is scaled between 0 and 1 that measures how strongly the peptide binds. NetMHCpan-4.1 was trained on BA data and additional eluted ligand (EL) data from mass spectrometry experiments from multiple sources (4). The EL data includes multi-allele information that was deconvoluted into single allele datapoints using NNAlign_MA. EL score is binary (either 0 or 1) since it checks if a peptide is present in a MHC’s immunopeptidome. The combined BA and EL dataset contained more than 13 million pMHC data points spread across numerous HLAs. For all our analyses, we focused on peptides of length 9, i.e. 9-mers, as these are the most frequent length of antigens in human immunopeptidomes. Also, we chose to analyze the 3 MHC molecules HLA-A02:01 (A2), HLA-B27:05 (B27), and HLA-B08:01 (B8). We picked these HLAs because they were highly represented in the training set (A2 ranked 1st, B27 ranked 11th, and B8 ranked 8th based on number of training datapoints), they are HLA supertypes (they represent the behavior of numerous less frequent HLA types), and they have different binding motifs (discussed in section 2.2).

**Figure 1:**
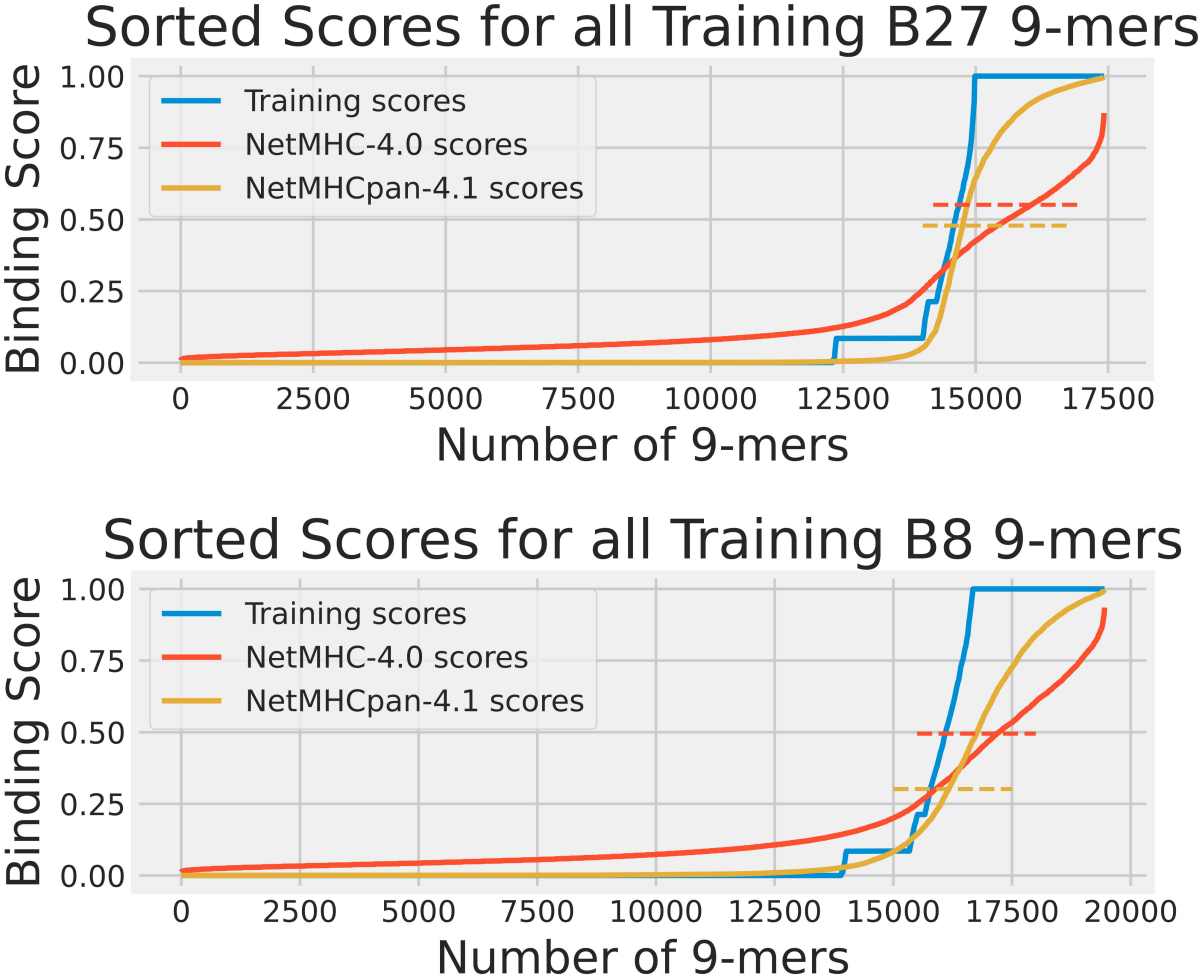
The cumulative distribution of the experimental training scores (blue), NetMHC-4.0 predicted scores (red), and NetMHCpan-4.1 predicted scores (yellow) for peptides in the training dataset for HLAs A2, B27, and B8. The strong binder thresholds for NetMHC-4.0 and NetMHCpan-4.1 are shown as dashed lines of the corresponding colors. For B27, these were 0.551 and 0.478, and for B8 these were 0.495 and 0.301 respectively. Each plot of scores was independently sorted. Consequently, the order of peptides is not conserved across the 3 plots in each subfigure. Note that the A2 results can be accessed from our previous study (15). For A2, the NetMHC-4.0 and NetMHCpan-4.1 thresholds were 0.659 and 0.419 respectively.

Our first analysis was the training data analysis. For each HLA, we collected all its 9-mers that were reported in the training dataset. A2 had 52569 9-mers, B27 had 17422, and B8 had 19448. The distributions of the experimentally obtained training scores for these HLAs are shown in **Figure 1**. We ran NetMHC-4.0 and NetMHCpan-4.1 on these 9-mers to gather each neural network’s predicted binding scores with the corresponding HLAs. These scores are shown in **Figure 1**. Furthermore, both tools classified 9-mers with large enough predicted binding scores (using a 0.5% rank to be precise) as strong binders for a tested HLA. We measured these thresholds by finding the lowest predicted binding score for a strong binder identified by these tools. **Figure 1** also shows these measured thresholds. Please note that this component of the training data approach had been used in our previous publication (15) for analyzing A2, so only B27 and B8 results are shown in this Figure.

As the training scores were available for all 9-mers we tested in the training data analysis, we also calculated confusion matrices (i.e. we counted the number of positives and negatives, both true and false) for both neural network tools. We used the previously measured strong binding thresholds on the actual training scores for each 9-mer to identify actual strong binders and non-binders in the context of each neural network tool. For example, in **Figure 1** all 9-mers on the blue plot above the red dashed line were classified as actual strong binders when testing NetMHC-4.0. The results of the confusion matrices are shown in **Table S1**. We also plotted the receiver operating characteristic (ROC) curve for each neural network tool for all 3 HLAs, as shown in **Figure S1**. We also computed the accuracy, precision, recall, and F1 score from the confusion matrices (17).

**Figure 2:**
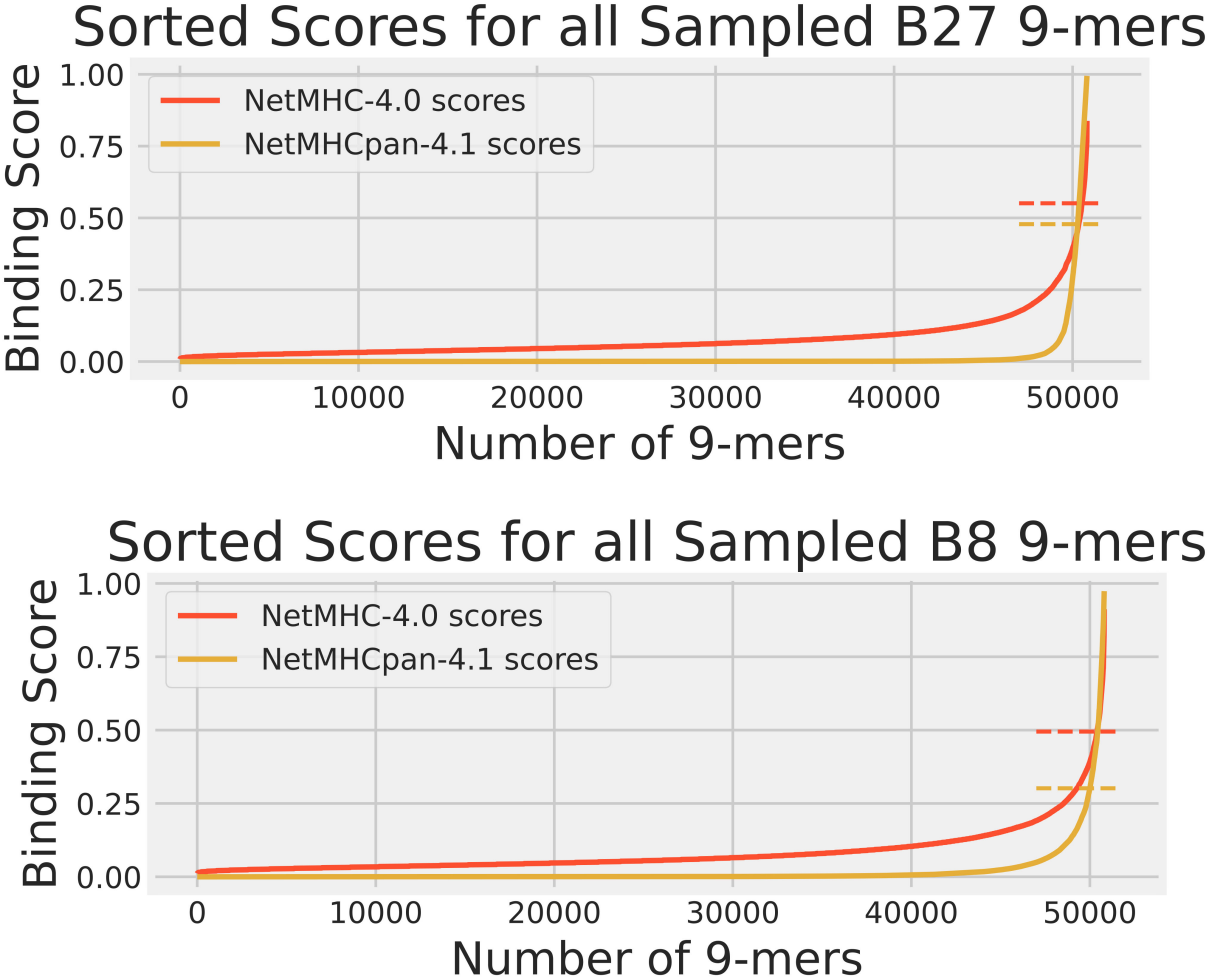
The cumulative distribution of NetMHC-4.0 predicted scores (red) and NetMHCpan-4.1 predicted scores (yellow) for peptides in the human proteome dataset for HLAs A2, B27, and B8. The strong binder thresholds for NetMHC-4.0 and NetMHCpan-4.1 are shown as dashed lines of the corresponding colors. These thresholds are the same as those in **Figure 1**. Each plot of scores was independently sorted. Consequently, the order of peptides is not conserved across the 2 plots in each subfigure. Note that the A2 results can be accessed from our previous study (15).

While the training data analysis was useful for identifying prediction biases, it alone was not a sufficient means for comparing NetMHC-4.0 and NetMHCpan-4.1. As NetMHC-4.0 was only trained on BA data while NetMHCpan-4.1 was trained on BA and EL data, NetMHCpan-4.1 had an advantage of having “seen” the EL peptides in its training over NetMHC-4.0. Therefore, we performed a human proteome analysis. We gathered the protein sequences for all reviewed human proteins from Uniprot (18), randomly sampled 100 of them, and fragmented them to create a set of 50804 9-mers. These peptides were also passed through NetMHC-4.0 and NetMHCpan-4.1 for all 3 HLAs to gather their predicted scores. These scores are shown in **Figure 2**. Since no experimentally obtained binding scores were available for these peptides, the Pearson correlations and the confusion matrices were not calculated. Again, this sampled human proteome approach had also been used in our previous publication (15) for analyzing A2 so only B27 and B8 results are shown here.

### 2.2 Hydrophobicity

As noted in section 2.1, one of the reasons we chose A2, B27, and B8 as our 3 target HLAs was their different binding motifs (19). A2 has a strong affinity for 9-mers with hydrophobic amino acids such as L, V, M, and I in positions 2 and 9. B27, on the other hand, binds 9-mers with hydrophilic R at position 2. In between these two, B8 prefers to bind 9-mers with both hydrophobic amino acids L, V, M, and I at position 2 and 9, but also hydrophilic amino acids R and K at position 3 and 5. Clearly hydrophobicity plays a crucial role in distinguishing the binding preferences of different HLAs. For our analyses, we decided to investigate the role of hydrophobicity in NetMHC-4.0’s and NetMHCpan-4.1’s predictions.

Hydrophobicity scales assign hydrophobicity values to single amino acids. They are designed so the hydrophobicity of long peptides or protein chains can be estimated by simply linearly adding up the scores of their constituent amino acids. Scales such as Kyte-Doolittle (20), Cornette (21), and Hopp-Woods  (22) are commonly used. However, we settled on the Moon scale  (23) for calculating hydrophobicity in our analyses as it specifically focuses on the sidechain hydrophobicity and polarity of single amino acids. Unlike the other scales, which are well suited for protein folding problems that do not correlate with sidechain hydrophobicity (24), the Moon scale is more representative of how small peptides would behave in an aqueous solution. The scale ranks the 20 amino acids in decreasing order of hydrophobicity as follows: F (1.43), L (1.26), I (1.15), P (1.13), Y (0.94), V (0.80), M (0.79), W (0.63), A (0.46), C (0.24), E (-0.27), G (-0.30), T (-0.33), S (-0.35), D (-0.85), Q (-0.88), N (-1.08), R (-1.19), H (-1.65), K (-1.93).

For any given 9-mer, we calculated its total hydrophobicity by adding up the Moon scale values for each of its 9 amino acids. For any given set of peptides, we measured the mean and standard deviation of the hydrophobicity scores of all peptides in it. Furthermore, we classified any given peptide into 1 of 3 classes: Hydrophobic (total hydrophobicity greater than 3), Hydrophilic (total hydrophobicity less than -3), or Balanced (total hydrophobicity between -3 and 3). This classification distinguished peptides based on their net hydrophobicity, and allowed us to investigate the impact of hydrophobicity on the differential prediction of MHC binding for different classes of peptides. We added these categories in **Figure S1** and **Tables S1** and **Table 1** to highlight any prominent trends specific to a peptide category.

**Table 1:** Various binary classification metrics on the training data analysis for NetMHC-4.0 (N-4.0) and NetMHCpan-4.1 (NP-4.1).

HLA	Peptide Case	Accuracy		Precision		Recall		F1 score	
		N-4.0	NP-4.1	N-4.0	NP-4.1	N-4.0	NP-4.1	N-4.0	NP-4.1
A2	All	0.918	0.936	0.882	0.918	0.618	0.756	0.727	0.829
	Hydrophobic only	0.875	0.871	0.861	0.922	0.745	0.735	0.745	0.818
	Hydrophilic only	0.986	0.991	0.786	0.826	0.134	0.613	0.229	0.704
	Balanced only	0.924	0.952	0.909	0.915	0.514	0.776	0.657	0.840
B27	All	0.909	0.965	0.914	0.917	0.460	0.865	0.612	0.890
	Hydrophobic only	0.926	0.969	0.954	0.964	0.535	0.832	0.685	0.893
	Hydrophilic only	0.916	0.957	0.793	0.832	0.362	0.814	0.497	0.823
	Balanced only	0.903	0.966	0.918	0.919	0.454	0.878	0.608	0.898
B8	All	0.927	0.951	0.925	0.915	0.625	0.818	0.746	0.864
	Hydrophobic only	0.915	0.935	0.910	0.930	0.571	0.728	0.702	0.816
	Hydrophilic only	0.960	0.965	0.921	0.892	0.728	0.832	0.813	0.861
	Balanced only	0.924	0.953	0.930	0.914	0.627	0.840	0.749	0.876

It was possible the smaller BA training dataset for NetMHC-4.0 was biased or unrepresentative of the numerous possible binding peptides. This bias could also be caused due to the binding affinity assays used to obtain BA scores, since these experiments only measure MHC-peptide affinity and do not account for the rest of the antigen presentation pathway or physiological conditions. Therefore, we also compared the hydrophobicities of the set of BA training data points (i.e. peptides NetMHC-4.0 was trained on) to the set of EL training data points (i.e. more than 80% of the peptides NetMHCpan-4.1 was trained on) for all 3 HLAs.

## 3 Results

From the scores shown in **Figure 1**, it was clear that the pMHC binding data fit a mostly binary data classification problem, since only 15% of the analyzed peptides had a training score not equal to 0 or to 1. This was mostly due to the addition of EL data which provided a binary “yes” or “no” answer to whether a given peptide was found attached to our chosen HLAs through mass spectroscopy. NetMHC-4.0’s predicted scores were dispersed smoothly between 0 and 1. In contrast, NetMHCpan-4.1 had more lopsided predictions with more non-binders assigned a binding score of 0. However, neither neural network tool predicted a definitive score of 1 to strong binders and instead used their thresholds to identify binders. NetMHCpan-4.1 predicted more strong binders than NetMHC-4.0 in all 3 cases. A2 results can be accessed from our previous publication (15).

These results of NetMHC-4.0 and NetMHCpan-4.1 on the sample human proteome are shown in **Figure 2**. Note that no experimental binding data was available for these peptides, and that the same set of these peptides was used for each HLA’s predictions when comparing **Figure 2** with 1. Again, NetMHCpan-4.1 seemed more stringent in predicting non-zero binding scores. NetMHCpan-4.1 also predicted slightly more strong binders than NetMHC-4.0 for all 3 HLAs.

The performances of NetMHC-4.0 and NetMHCpan-4.1 as binary classifiers are shown in **Figure S1** as ROC curves. The figure also includes the area under the curve (AUC) for each classifier. It breaks down the performance across all training peptides and even the 3 peptide categories defined in 2.2. For A2 and B27, NetMHC-4.0 and NetMHCpan-4.1 had similar AUC values (no more than 1% apart), while for B8 NetMHC-4.0 under-performed by 3%. Across all HLAs, both tools reported AUC values higher than 95%. The different peptide categories did not highlight any notable trends on the ROC plots. It is interesting to note that both tools had different performances across the 3 peptide cases in each HLA. To investigate this observation in detail, we used violin plots to visualize the predicted immunopeptidomes.

**Figure 3:**
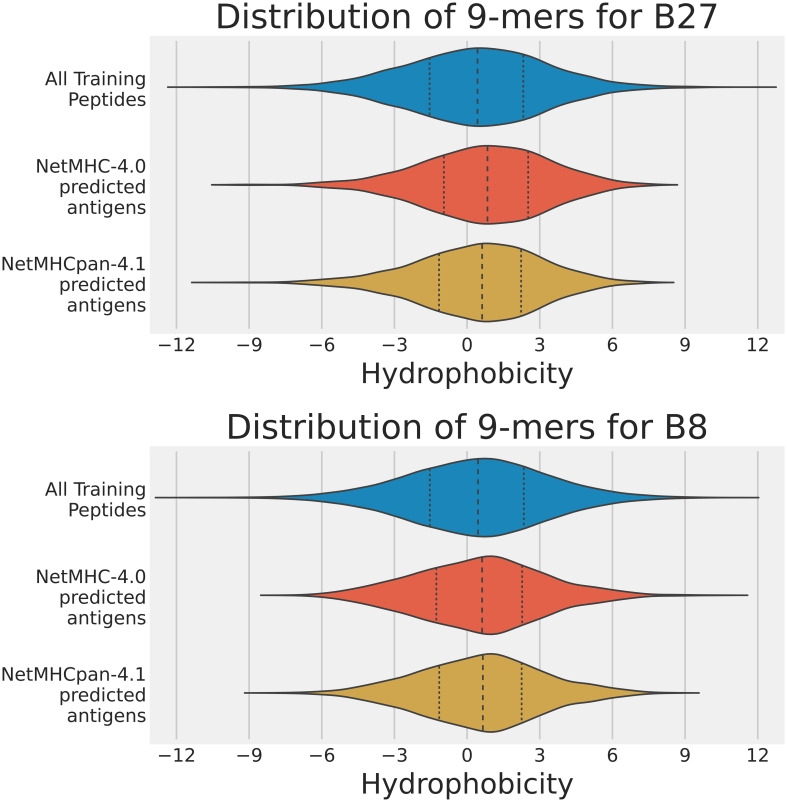
Violin plots of the hydrophobicity of the sets of strong binders predicted by NetMHC-4.0 and NetMHCpan-4.1 on the training dataset for A2, B27, and B8. The x-axis represents the hydrophobicity of a 9-mer, and the y-axis represents the frequency. Note that the A2 results can be accessed from our previous study (15). The mean and two quartiles are also depicted in each distribution.

**Figure 4:**
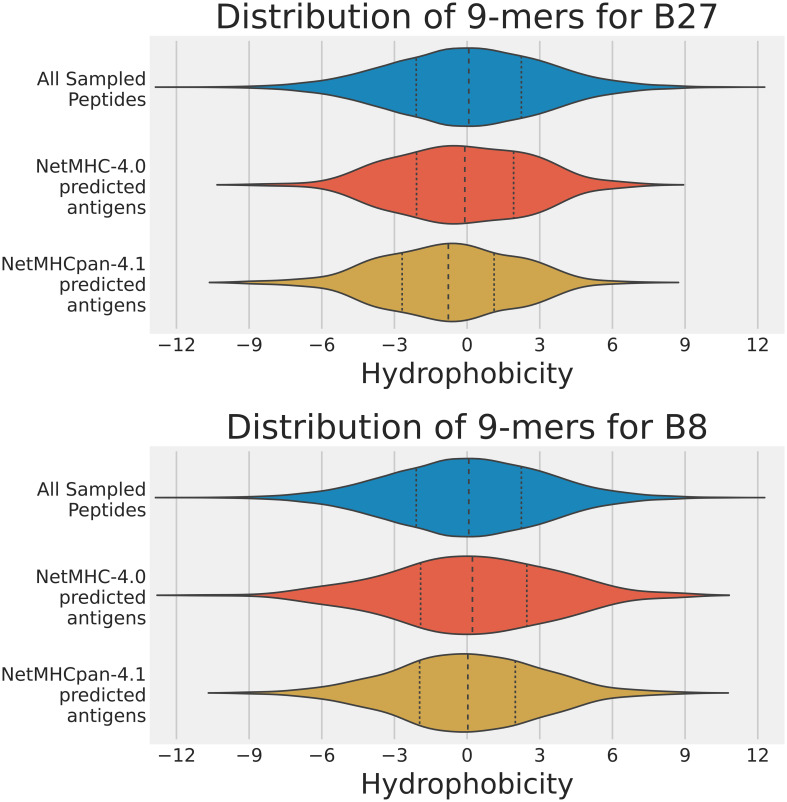
Violin plots of the hydrophobicity of the sets of strong binders predicted by NetMHC-4.0 and NetMHCpan-4.1 on the human proteome dataset for A2, B27, and B8. The x-axis represents the hydrophobicity of a 9-mer, and the y-axis represents the frequency. The distributions of all sampled peptides (blue), strong binders predicted by NetMHC-4.0 (red), and those predicted by NetMHCpan-4.1 (yellow) are shown. The mean and two quartiles are also depicted in each distribution. Note that the A2 results can be accessed from our previous study (15).

The distributions of the hydrophobicity scores of 9-mers in the training data analysis are shown in **Figure 3**, and those in the human proteome data analysis in **Figure 4**. For both analyses, we used the 2 sample t-test to compare the immunopeptidomes predicted by NetMHC-4.0 and NetMHCpan-4.1, and to identify any discrepancies in their predictions on the basis of hydrophobicity (all values used in the t-tests are listed in **Table S2**).

Strong binders to A2 were expected to have two hydrophobic amino acids (L, V, M, or I) at positions 2 and 9, and thus the expected A2 immunopeptidome would be more hydrophobic than the training or sampled data (expected to be approximately centered about a Moon score of 2). In both analyses, NetMHCpan-4.1 predicted strong binders with a closer hydrophobicity score to our expected value than NetMHC-4.0 did. This difference in predictions was extremely statistically significant in both analyses (p-values less than 0.0001). That is, NetMHC-4.0’s predicted strong binders for A2 were more hydrophobic than NetMHCpan-4.1’s.

Strong binders to B27 were expected to have one hydrophilic amino acid (R) at position 2, and thus the expected B27 immunopeptidome would be slightly more hydrophilic than the training or sampled data (expected to be approximately centered about a Moon score of -1). Neither tool exhibited this hydrophilic shift in its predictions on the training dataset, but with the human proteome, NetMHCpan-4.1 did predict strong binders centered at a hydrophobicity score of -0.775; NetMHC-4.0’s mean was -0.155. The difference in predictions was statistically significant in both analyses (p-values no larger than 0.002). Again, NetMHC-4.0’s predicted strong binders for B27 were more hydrophobic than NetMHCpan-4.1’s.

Strong binders to B8 were expected to have two hydrophobic amino acids (L, V, M, or I) at positions 2 and 9, and two hydrophilic amino acids (R or K) at positions 3 and 5. Consequently, no major shift in hydrophobicity expected in the B8 immunopeptidomes predicted by either neural network tool. This was indeed the result observed in both analyses, and no significant difference was observed between the predictions of NetMHC-4.0 and NetMHCpan-4.1 (p-values were 0.716 and 0.425 for the training dataset analysis and the human proteome analysis respectively). In this case, NetMHC-4.0’s predicted set of binders for B8 were not distinguishable from NetMHCpan-4.1’s in terms of hydrophobicity.

Overall, the trend observed from these violin plots seemed to be that NetMHC-4.0 was incorrectly accounting for hydrophobicity when predicting strong binders for A2 and B27. In contrast, NetMHCpan-4.1 was predicting less hydrophobic strong binders for allHLAs in the human proteome analysis. As NetMHCpan-4.1 more closely matched our expectations for A2 and B27, we reasoned that its predictions were more accurate. Since NetMHCpan-4.1 also predicted more strong binders, we hypothesized that the new strong binders gained in NetMHCpan-4.1’s immunopeptidome were slightly hydrophilic (with respect to NetMHC-4.0’s immunopeptidome) and therefore skewing the mean hydrophobicity lower. To investigate this, we referred to **Table 1** in which we tracked each neural network tool’s performance on the training data. In particular, we broke down the performances of these tools in our 3 specific peptide cases using 4 different classification metrics discussed below.

The Accuracy metric tracks the number of true negatives and true positives identified by a classifier relative to all the tested data points. Across all HLAs, both neural network tools maintained high accuracy, though NetMHCpan-4.1 performed slightly better (by roughly 2%). For B27 in particular, NetMHCpan-4.1 had a notably higher accuracy (by about 6%) in all peptides cases. No specific improvement was observed in any individual peptide category.

The Precision metric inversely measures the number of false positives identified by a classifier. NetMHCpan-4.1 exhibited higher precision for A2 (by 3%), roughly equivalent precision for B27, and slightly lower precision for B8 (by about 2%) in all peptide cases. The highlight here was that NetMHC-4.0 had low precision (lower than 80%) when dealing with hydrophilic peptides for A2 and B27.

The Recall metric inversely represents the number of false negatives not identified by a classifier. NetMHCpan-4.1 showed significant improvement (consistently higher than 10%) in recall for all HLAs. For A2, these improvements were observed in classifying hydrophilic and balanced peptides. For B27 and B8, these improvements were observed in all peptide categories.

The F1 score is a combination of precision and recall, and tracks overall performance of a classifier. For all HLAs, NetMHCpan-4.1 outperformed NetMHC-4.0 (by at least 10%) when considering all peptides categories.

The observations from these data, in particular the accuracy and F1 score, support our initial assumption that NetMHCpan-4.1 had stronger predictions than NetMHC-4.0 when focusing on hydrophobicity. The precision and recall scores elucidate the reasons behind this improvement: NetMHCpan-4.1 predicted fewer false positives, and much fewer false negatives for all HLAs. The sources of these false positives and negatives in NetMHC-4.0’s predictions varied across the different HLAs. For A2, most false positives were found in non-balanced (hydrophobic and hydrophilic) peptides cases, while the majority of false negatives came from non-hydrophobic peptides. For B27, a few false positives were observed in the hydrophilic peptides, but most notably numerous false negatives were found across all types of peptides. For B8, false negatives in all peptides cases lowered the performance of NetMHC-4.0.

It is also important to acknowledge that NetMHCpan-4.1 has an unfair advantage over NetMHC-4.0 – the newer tool was trained on a much larger dataset. Furthermore, NetMHC-4.0 was trained on only peptide-MHC binding affinity data, while NetMHCpan-4.1 was trained on eluted ligand data that was representative of the entire antigen presentation pathways. We investigated the possibility of the small BA data in the training dataset being biased towards being hydrophobic. These values were contrasted to the mean hydrophobicity values of the EL dataset in **Figure S2**. In each case, the BA training data was more hydrophobic than the EL training data set. This bias was the most prominent in the A2 peptides, and least prominent in B27. This discrepancy in training data could be one of the causes for why NetMHC-4.0’s predicted strong binders contained many hydrophilic false negatives.

## 4 Conclusion

In our previous study, we had identified a significant preference for hydrophobic peptides in NetMHC-4.0’s predicted immunopeptidome for A2 (15). We had argued that highly hydrophobic peptides were being classified by NetMHC-4.0 as false positives. We had suggested that highly hydrophobic peptides would never be trafficked in the aqueous cytosol of cells and were therefore obvious false positives.

In this study, we expanded our previous research to focus on more HLA types – i.e. A2, B27, and B8. These HLAs prefer to bind hydrophobic, hydrophilic, and balanced (neither hydrophobic nor hydrophilic) peptides respectively. By comparing the predictions by NetMHC-4.0 and NetMHCpan-4.1 on both the training dataset (see **Figure 3**) and the sampled human proteome (see **Figure 4**), we confirmed NetMHC-4.0’s hydrophobicity bias for A2 and B27. In these cases, NetMHC-4.0’s predicted immunopeptidome was much more hydrophobic than NetMHCpan-4.1’s predictions. This hydrophobic bias was not statistically significant in the B8 immunopeptidome. These results suggest that NetMHC-4.0 struggles to predict strong binders correctly in HLAs with strong hydrophobic or hydrophilic binding motifs.

We used several machine learning metrics, such as accuracy, and recall, on the training dataset analysis (see **Table 1**). From these results, we discovered the improvement in NetMHC-4.0’s predictions (over NetMHC-4.0’s) stemmed from fewer false negatives in the non-balanced peptide cases, and fewer false positives in general. In particular, the biased immunopeptidome predicted by NetMHC-4.0 was not just a consequence of overestimating the binding of hydrophobic peptides, but also due to overlooking binders that were hydrophilic.

A key takeway of our analyses is that this hydrophobicity bias could only be discovered and expounded upon by focusing on hydrophobicity of peptides as a core factor in pMHC binding. Merely using machine learning metrics without accounting for such biochemical attributes would have been insufficient in capturing this bias. This is evident from how both neural network tools had similar performances across all 3 HLAs in **Figure S1**. Just as understanding the erroneous predictions from NetMHC-4.0 required the use of hydrophobicity as a metric, we believe that mechanistically modelling the biochemistry (to some extent) improves upon a purely data-driven artificial intelligence’s prediction.

We conclude that NetMHCpan-4.1 is the more reliable of the two neural network tools. It had stronger results across the various metrics we used, and the hydrophobicity of its predicted immunopeptidome matched our expected hydrophobicity values. In contrast, NetMHC-4.0 struggled to predict all strong binders for HLAs that had notable hydrophobic or hydrophilic preferences. There could be several reasons why NetMHCpan-4.1 outperformed NetMHC-4.0. NetMHCpan-4.1 had a larger training set. Binding Affinity data alone could not model the effects of the entire antigen presentation pathway as Eluted Ligand data could have. For example, the binary values of eluted ligand data might have trained NetMHCpan-4.1 to be more decisive in its predictions. Tracking the MHC sequence could have allowed NetMHCpan-4.1 to model binding mechanics of MHC binding pockets. Eluted ligand data might have set NetMHCpan to capture aspects of the entire antigen presentation pathway instead of estimating pMHC binding strength alone. The generation of negative training data  (16) for NetMHCpan-4.1 could have resolved false positives that NetMHC-4.0 was vulnerable to.

In future work, we will focus on identifying more significant structural and mechanistic attributes that pose hurdles for AI-based methods. We are developing a structural prediction tool capable of predicting peptide binding with uncurated MHC molecules. Since we were limited to using the training dataset, and the sample human proteome dataset without binding data, it would be interesting to expand upon this study with a large evaluation dataset to test the predictions of NetMHCpan-4.1 and NetMHC-4.0 as well.

## Data availability statement

The original contributions presented in the study are included in the article/**Supplementary Materials**. Further inquiries can be directed to the corresponding author.

## Author contributions

AS, MR, JC, JU, and GV led discussions on this research. AS conducted the data analyses and wrote the manuscript. AS, MR, and GV reviewed the manuscript. All authors contributed to the article and approved the submitted version.

## Funding

This research was funded by the NSF Grant 2036064.

## Acknowledgments

We thank Chad Myers for recommending using machine learning metrics to score neural networks.

## Conflict of interest

The authors declare that the research was conducted in the absence of any commercial or financial relationships that could be construed as a potential conflict of interest.

## Publisher’s note

All claims expressed in this article are solely those of the authors and do not necessarily represent those of their affiliated organizations, or those of the publisher, the editors and the reviewers. Any product that may be evaluated in this article, or claim that may be made by its manufacturer, is not guaranteed or endorsed by the publisher.

## Supplementary material

Supplementary Figure 1 Receiver Operating Characteristic curves for NetMHC-4.0 (left) and NetMHCpan-4.1 (right) for A2, B27, and B8 based on the training dataset. In each subfigure, plots are drawn for all peptides (blue), hydrophobic peptides only (red), hydrophilic peptides only (yellow), and balanced peptides only (green). A completely random classifier is also plotted for reference (dashed black). For each plot, the Area Under the Curve (AUC) is also noted in the legend.

Supplementary Figure 2 Violin plots of the hydrophobicity of all the training 9-mers, split based on experimental source: Binding Affinity (in blue) and Eluted Ligand (in red). For A2, there were 7940 BA peptides and 44719 EL peptides, with mean hydrophobicities of 2.227 and 0.667 respectively. For B27, there were 2683 BA peptides and 14739 EL peptides, with mean hydrophobicities of 0.597 and 0.322 respectively. For B8, there were 2777 BA peptides and 16671 EL peptides, with mean hydrophobicities of 0.999 and 0.292 respectively.
