# Supplementary material for: Hydrophobicity identifies false positives and false negatives in peptide-MHC binding
Source: Front Oncol. 2022 Nov 7;12:1034810. doi: 10.3389/fonc.2022.1034810 (PMC9677119; doi:10.3389/fonc.2022.1034810)
Supplement: Supplementary Figure 1 — Receiver Operating Characteristic curves for NetMHC-4.0 (left) and NetMHCpan-4.1 (right) for A2, B27, and B8 based on the training dataset. In each subfigure, plots are drawn for all peptides (blue), hydrophobic peptides only (red), hydrophilic peptides only (yellow), and balanced peptides only (green). A completely random classifier is also plotted for reference (dashed black). For each plot, the Area Under the Curve (AUC) is also noted in the legend. [file DataSheet_1.pdf]

Supplementary Material

The changes in predictions observed in Figure 1 suggest that NetMHCpan-4.1 approximated the training scores better than NetMHC-4.0 did in Figure 1 (the yellow plot fitted the S-curve transition of the blue plot more tightly than the red plot did). To confirm this, we calculated the Pearson Correlation of the training data with NetMHC-4.0, and with NetMHCpan-4.1. The correlation coefficients for NetMHC-4.0 and NetMHCpan-4.1 for A2 were 0.8492 and 0.8637 respectively. For B27, these coefficients were 0.8165 and 0.8844. For B8, these were 0.8492 and 0.863. It is likely that the stronger correlation for NetMHCpan-4.1 is a consequence of NetMHCpan-4.1 having "seen" EL peptides which NetMHC-4.0 would not have been trained on.

Table S1. Confusion matrices for the training data analysis for NetMHC-4.0 and NetMHCpan-4.1. We counted True Negatives (TN), False Positives (FP), False Negatives (FN), and True Positives (TP).

HLA	Peptide Case	NetMHC-4.0				NetMHCpan-4.1			
		TN	FP	FN	TP	TN	FP	FN	TP
A2	All	42621	770	3540	5728	41166	730	2630	8133
	Hydrophobic only	8050	516	1094	3199	7467	316	1345	3731
	Hydrophilic only	5213	3	71	11	5193	12	36	57
	Balanced only	29316	250	2371	2508	28464	402	1247	4332
B27	All	14573	118	1476	1255	14367	221	384	2450
	Hydrophobic only	2581	12	215	247	2564	15	80	396
	Hydrophilic only	1946	24	162	92	1905	45	51	223
	Balanced only	10028	82	1098	914	9880	161	253	1828
B8	All	15923	169	1257	2099	15495	280	667	3006
	Hydrophobic only	2864	35	265	353	2785	38	189	505
	Hydrophilic only	2101	18	78	209	2058	32	53	263
	Balanced only	10950	116	913	1534	10645	210	424	2234

Table S2. Sizes, Means, and Standard Deviations of the sets of peptides reported in all Violin plots.The abbreviations used here are: all Training Peptides (TP), NetMHC-4.0 predicted Strong Binders (N-4.0 SB), NetMHCpan-4.1 predicted Strong Binders (NP-4.1 SB), and all Sampled Peptides (SP).

HLA	Value of Set	Training Data Analysis			Human Proteome Analysis		
		TP	N-4.0 SB	NP-4.1 SB	SP	N-4.0 SB	NP-4.1 SB
A2	Size	52659	6498	8863	50804	486	730
	Mean	0.902	3.458	2.756	0.052	4.519	2.789
	Std. Dev.	3.063	2.365	2.426	3.212	2.517	2.647
B27	Size	17422	1373	2671	50804	290	528
	Mean	0.364	0.725	0.450	0.052	-0.155	-0.775
	Std. Dev.	2.923	2.593	2.593	3.212	2.740	2.729
B8	Size	19448	2268	3286	50804	411	801
	Mean	0.393	0.544	0.570	0.052	0.182	0.031
	Std. Dev.	2.946	2.652	2.566	3.212	3.332	3.011

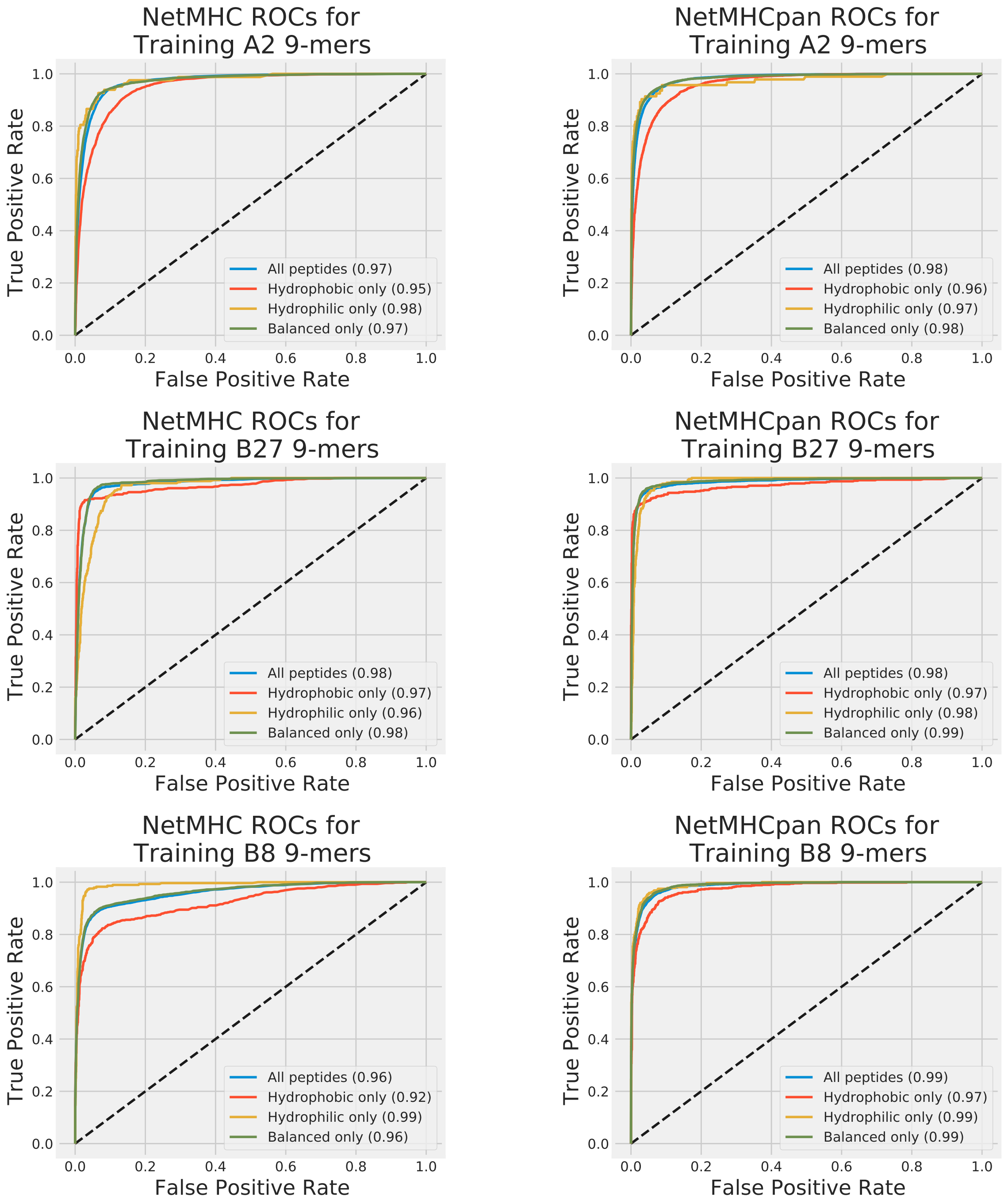
NetMHC ROCs for Training A2 9-mers
True Positive Rate
False Positive Rate
All peptides (0.97)
Hydrophobic only (0.95)
Hydrophilic only (0.98)
Balanced only (0.97)
NetMHCpan ROCs for Training A2 9-mers
All peptides (0.98)
Hydrophobic only (0.96)
Hydrophilic only (0.97)
Balanced only (0.98)
NetMHC ROCs for Training B27 9-mers
All peptides (0.98)
Hydrophobic only (0.97)
Hydrophilic only (0.96)
Balanced only (0.98)
NetMHCpan ROCs for Training B27 9-mers
All peptides (0.98)
Hydrophobic only (0.97)
Hydrophilic only (0.98)
Balanced only (0.99)
NetMHC ROCs for Training B8 9-mers
All peptides (0.96)
Hydrophobic only (0.92)
Hydrophilic only (0.99)
Balanced only (0.96)
NetMHCpan ROCs for Training B8 9-mers
All peptides (0.99)
Hydrophobic only (0.97)
Hydrophilic only (0.99)
Balanced only (0.99)
0.0
0.2
0.4
0.6
0.8
1.0

Figure S1.

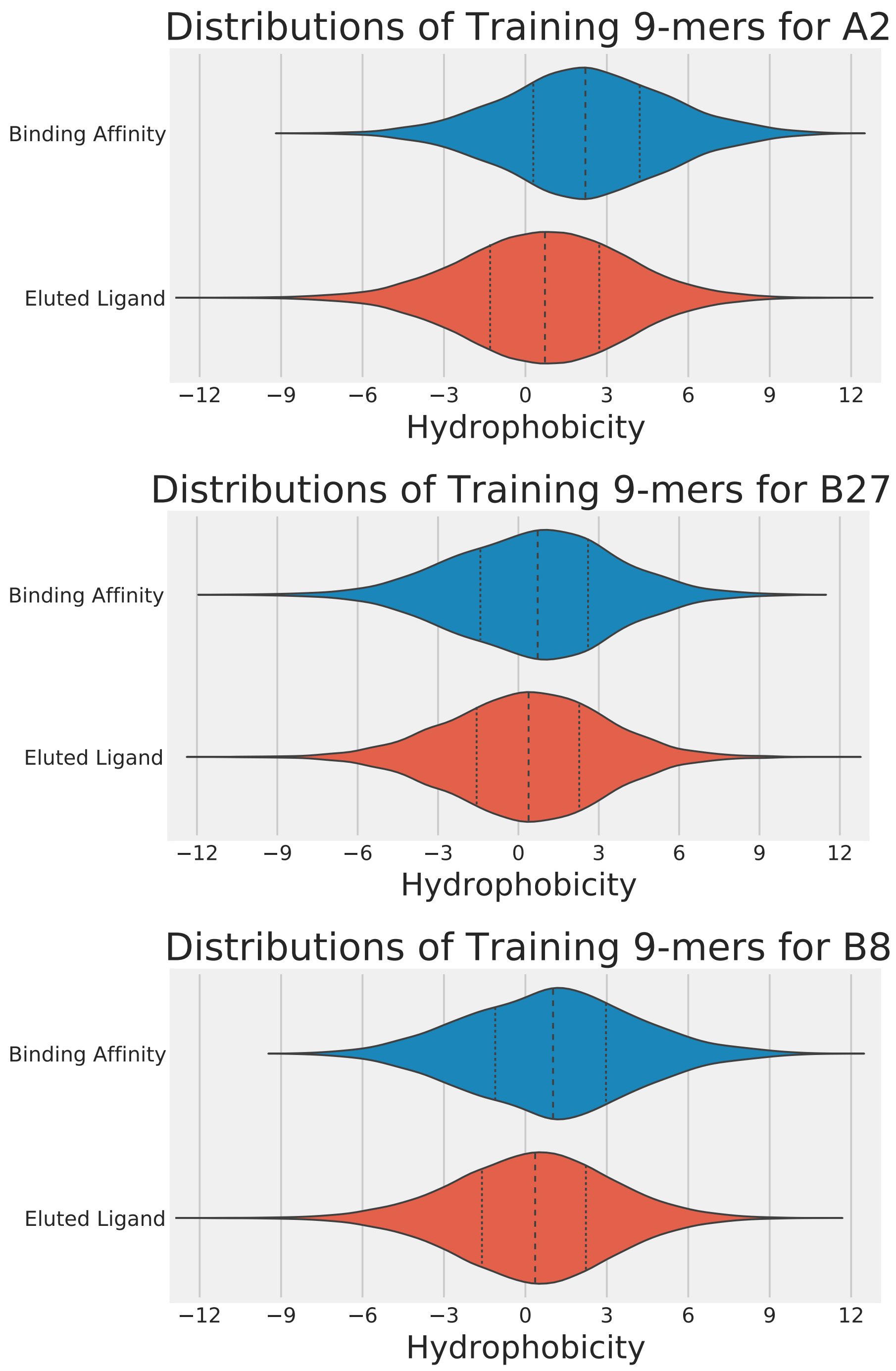
Distributions of Training 9-mers for A2
Binding Affinity
Eluted Ligand
−12 −9 −6 −3 0 3 6 9 12
Hydrophobicity
Distributions of Training 9-mers for B27
Binding Affinity
Eluted Ligand
−12 −9 −6 −3 0 3 6 9 12
Hydrophobicity
Distributions of Training 9-mers for B8
Binding Affinity
Eluted Ligand
−12 −9 −6 −3 0 3 6 9 12
Hydrophobicity

Figure S2.
